# A Pilot Study on Brain Plasticity of Functional Connectivity Modulated by Cognitive Training in Mild Alzheimer’s Disease and Mild Cognitive Impairment

**DOI:** 10.3390/brainsci7050050

**Published:** 2017-04-29

**Authors:** Francesco Barban, Matteo Mancini, Mara Cercignani, Fulvia Adriano, Roberta Perri, Roberta Annicchiarico, Giovanni Augusto Carlesimo, Claudia Ricci, Maria Giovanna Lombardi, Valeria Teodonno, Laura Serra, Giovanni Giulietti, Lucia Fadda, Alessia Federici, Carlo Caltagirone, Marco Bozzali

**Affiliations:** 1 Clinical and Behavioral Neurology Laboratory, IRCCS Santa Lucia Foundation, Rome 00179, Italy; f.adriano@hsantalucia.it (F.A.); r.perri@hsantalucia.it (R.P.); r.annicchiarico@hsantalucia.it (R.A.); memolab@hsantalucia.it (G.A.C.); c.ricci@hsantalucia.it (C.R.); mg.lombardi@hsantalucia.it (M.G.L.); vteodonno@gmail.com (V.T.); l.fadda@hsantalucia.it (L.F.); a.federici@hsantalucia.it (A.F.); c.caltagirone@hsantalucia.it (C.C.); 2 Neuroimaging Laboratory, IRCCS Santa Lucia Foundation, Rome 00179, Italy; l.serra@hsantalucia.it (L.S.); g.giulietti@hsantalucia.it (G.G.); m.bozzali@hsantalucia.it (M.B.); 3 Humboldt-Universität zu Berlin, Berlin School of Mind and Brain, Berlin 10117, Germany; 4 Department of Engineering, University of Rome “Roma Tre”, Rome 00146, Italy; ingmatteomancini@gmail.com; 5 Clinical Imaging Sciences Centre, Brighton and Sussex and Medical School, Brighton BN1 9RR, UK; m.cercignani@bsms.ac.uk; 6 Department of Systemic Medicine, University of Tor Vergata, Rome 00173, Italy

**Keywords:** Alzheimer’s disease, mild cognitive impairment, neural plasticity, DMN, connectomics, fMRI, graph theory, cognitive training

## Abstract

Alzheimer’s disease (AD) alters the functional connectivity of the default mode network (DMN) but also the topological properties of the functional connectome. Cognitive training (CT) is a tool to slow down AD progression and is likely to impact on functional connectivity. In this pilot study, we aimed at investigating brain functional changes after a period of CT and active control (AC) in a group of 26 subjects with mild AD (mAD), 26 with amnestic mild cognitive impairment (aMCI), and a control group of 29 healthy elderly (HE) people. They all underwent a CT and AC in a counterbalanced order following a crossover design. Resting-state functional MRI and neuropsychological testing were acquired before and after each period. We tested post-CT and post-AC changes of cognitive abilities, of the functional connectivity of the DMN, and of topological network properties derived from graph theory and network-based statistics. Only CT produced functional changes, increasing the functional connectivity of the posterior DMN in all three groups. mAD also showed functional changes in the medial temporal lobe and topological changes in the anterior cingulum, whereas aMCI showed more widespread topological changes involving the frontal lobes, the cerebellum and the thalamus. Our results suggest specific functional connectivity changes after CT for aMCI and mAD.

## 1. Introduction

Alzheimer’s disease (AD) is the most common form of dementia, characterized by a progressive impairment of memory and executive functions. It is expected to increase in the next decades, thus becoming a public emergency [1].

A combination of atrophy of the medial temporal lobes and disconnections between brain regions underlies the progressive clinical evolution of the disease [2]. Critical medial brain regions for AD progression and brain resilience to disease are part of the so-called default mode network (DMN) comprising the precuneus, the posterior and anterior cingulum, the medial ventral prefrontal cortex, the inferior parietal cortex, and the medial temporal lobes [3,4,5]. This is a large-scale set of spatially distributed, but functionally co-activated, brain regions overlapping with areas that showed depositions of AD biomarkers [6,7], and it underlies complex cognitive functions such as memory processes [8]. Other authors have suggested a more widespread involvement affecting multiple networks in AD pathology [9]. Consistently, AD has been described as a disconnection syndrome [10]. Given that large-scale network changes underlie cognitive dysfunctions in AD, a more appropriate multivariate approach should be adopted to capture the negative brain plasticity effects of AD pathology [11] as well as the positive plasticity promoted by the restorative effects.

From a therapeutic point of view, only pharmacological symptomatic treatments are available, often resulting in modest beneficial effects with potential side effects [12]. Therefore, early diagnostic biomarkers and effective treatments are urgently needed. In this regard, in recent years non-pharmacological treatments have gained momentum. Recent neuroscientific evidence stimulated the development of effective cognitive training (CT) protocols, suggesting the presence of significant neuronal plasticity in the adult brain [13] supporting brain resilience against aging or even pathology. CT consists in the repeated practice, usually on a computer, of standard exercises focused on cognitive functions such as memory, executive functions, and language. Several studies showed that computerized CT promotes cognitive improvement in healthy elderly and in people with Mild Cognitive Impairment (MCI), a risk factor for dementia [14,15,16,17], whereas their effectiveness in AD patients is still controversial.

Brain plasticity effects promoted by CT in AD [18] and MCI [19] have been previously shown by comparing brain activations during the execution of a task (i.e., task-based) before and after a period of CT. These studies mainly showed increased brain activation in regions of the DMN, but also in subcortical and dorsal regions, and one study also reported increased functional connectivity [20]. A recent, promising method to study functional connectivity consists of recording brain activity at rest with resting-state functional MRI (RS-fMRI), or other techniques such as EEG, when subjects are not executing any particular cognitive task. Previous evidence demonstrated that synchronous fluctuations of functional signal occur in different brain regions, believed to represent functional resting-state networks, and corresponding to neuronal activations during specific cognitive tasks [21]. Indeed, a previous study [22] recording brain activity at rest with an electroencephalogram (EEG) in a large sample of MCI before and after an intervention combining CT and physical activity showed a decreased activity of the posterior DMN.

Studies on healthy subjects suggest that, in order to understand the complex reorganizations of the brain after interventions, a higher level of organization should be taken into account [17]. Several studies on healthy subjects showed widespread changes after interventions involving functional connectivity within and among networks such as the DMN, salience, and executive networks related to the enhancement of behavioral outcomes [17,23,24]. Changes of functional connectivity promoted by CT might be detected in the strength of the coupling between pairs of brain regions [25], in the strength of the functional connectivity of networks of segregated but functionally co-activated regions such as the DMN, or in local or global topological properties of the whole brain, so-called connectomics [26]. The connectome is a description of the whole brain connectivity by means of graph theory and network modeling. It can be reconstructed with EEG or MRI data, where brain regions represent the nodes of the networks and functional connectivity methods are used to trace out the links between them. In particular, a useful approach is given by graph analysis [27], which assesses local and global topological properties of brain networks and their integrative and segregative characteristics. Global metrics describe how well clusters of nodes of the network are segregated, how integrated the network is, and how efficient the network is considering the quality of the processing of local information and the integration of distant local clusters. Local metrics describe the importance of a node in serving as a hub in the network. A recent review [28] reported several studies demonstrating that AD pathology disrupts global properties, showing small-worldness reduction [29], and local properties, showing functional impairment of the hubs within the DMN for both MCI and AD [30]. Moreover, a pioneering study of Klados and collaborators [31] showed that an intervention of combined physical and CT induced changes in the interregional functional connectivity of the whole brain of people with MCI.

The aim of the present study is to measure brain plasticity changes in a sample of 26 mild AD (mAD) patients, 26 amnestic MCI (aMCI) patients, and a control group of 29 healthy elderly (HE) people. The samples underwent a three-month period of computerized CT aimed at improving memory and executive function abilities, validated in our preliminary behavioral study [32]. We used a crossover design in which participants underwent, in a counterbalanced order, a three-month intervention of CT and a period of active control condition (AC) of the same length and intensity. Cognitive performance, assessed via standardized neuropsychological (NPS) tests assessing memory, executive functions and attention and RS-fMRI, was measured before and after each period. Our main hypothesis was that only the CT, and not AC, would produce changes in the functional connectivity between key regions for memory and executive function abilities affected by AD pathology. We also hypothesized that CT might show widespread effects involving the whole brain connectome.

For this reason, in the present study we investigated in mAD and aMCI patients and in HE:

(1) Cognitive changes, measured with NPS tests, after a period of CT vs. a period of AC in memory, executive functions and attentional abilities;
(2) Functional brain changes of the DMN, measured with RS-fMRI, after a period of CT vs. a period of AC;
(3) Functional connectomics brain changes, measured with RS-fMRI, in the coupling between pairs of brain regions of the whole brain and in global and local topological properties of large-scale brain networks through graph theoretical approach after a period of CT and, separately, after a period of AC.

## 2. Methods

### 2.1. Participants

**Figure 1:**
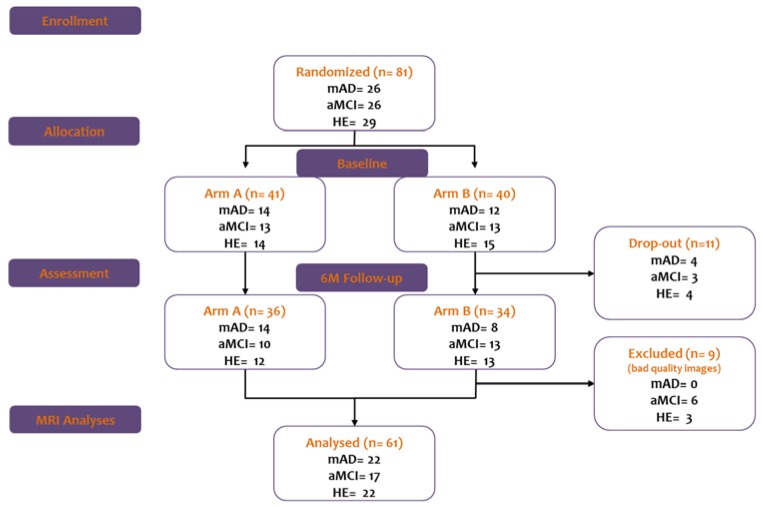
Workflow of the study showing the number of participants of each different group (mAD = mild Alzheimer’s Disease; aMCI = amnestic mild cognitive impairment; HE = healthy elderly) randomized in the two arms of the study. Arm A, in which participants first underwent the cognitive training period and then the active control condition period; arm B, in which participants first underwent the active control condition period and then the cognitive training period.

This longitudinal study involved 81 elderly Italian right-handed [33] subjects (age ≥ 60 years) (26 mAD, 26 aMCI, and 29 HE). Eleven subjects dropped out (4 mAD, 3 aMCI, and 4 HE) and nine were excluded for the low quality of images (6 aMCI and 3 HE) (Figure 1; Table 1).

Participants were included in the study according to the following criteria:

(1) mAD were consecutively recruited from the Dementia Clinic of the Santa Lucia Foundation in Rome (Italy) after they underwent a neurological assessment, a MRI examination to exclude the presence of macroscopic brain abnormalities, and an extensive NPS examination. The latter consisted of standardized tests assessing all major cognitive domains: verbal and visuospatial memory with the Rey Auditory Verbal Learning Test (RAVLT) [34] and the Rey–Osterrieth Complex Figure Test (ROCF) [35]; attention with Trail Making Test version A (TMT-A) [36], Digit Span Forward [37], Attentive Matrices [38]; executive functions with Phonological Word Fluency test [34], Clock Drawing Test [39], Trail Making Test version B-A (TMT B-A) [36], Wisconsin Card Sorting Test (WCST) [40]; language with Naming test [41]; reasoning with Colored Progressive Matrices [34]; constructional praxis with the copy of the ROCF; independent living with Instrumental Activities of Daily Living scale (IADL) [42]. Inclusion criteria were to meet the criteria for diagnosis of probable AD defined by the National Institute of Neurological and Communicative Disorders and Stroke (NINCDS) and the Alzheimer’s Disease and Related Disorders Association (ADRDA) [43] (confirmed with a follow-up neurological and NPS evaluation at the end of the study), a Mini-Mental State Examination (MMSE) [44] score ≥20, and a global score on the Clinical Dementia Rating (CDR) scale [45] equal to 1, with a sub-score of at least 1 in the memory domain. They all received a stable anti-dementia pharmacological treatment (for at least one month before the beginning of study and during the study period) [46]. Twenty-two mAD subjects were included in the behavioral and DMN analyses and 20 in the graph theory and NBS analyses.

(2) aMCI subjects were consecutively recruited from the Dementia Clinic of the Santa Lucia Foundation in Rome (Italy) and underwent the aforementioned neurological and NPS evaluation and MRI. Inclusion criteria were to meet the current operational criteria for amnestic MCI [47] for memory single- (*n* = 24) or memory multiple- (*n* = 2) domain (confirmed by a follow-up neurological and NPS evaluation at the end of the study), a MMSE score ≥25, and a global score on the CDR equal to 0.5, with a sub-score of 0.5 in the memory domain. Twenty-three aMCI subjects were included in the behavioral analyses and 17 in all the neuroimaging analyses.

(3) A control group of HE was enrolled from municipality senior centers of Rome. They all underwent the aforementioned extensive NPS evaluation to exclude the presence of cognitive deficits and a MRI examination to exclude the presence of macroscopic brain abnormalities. Inclusion criteria were absence of cognitive deficits, cerebro-vascular pathology or brain abnormalities, previous major systemic, psychiatric and/or neurological illness, a MMSE score ≥26, and a global score on the CDR equal to 0. Twenty-five HE subjects were included in the behavioral analyses, 22 in the DMN analyses, and 19 in the graph-theory and NBS analyses.

We also assessed the medial temporal lobe atrophy (MTA) in all participants by a visual assessment scale [48]. The scale provides a score between 0 (no atrophy) and 4 (severe volume loss of the medial temporal lobe) and scores ≥1.5 indicating a significant atrophy [49]. In particular, it was used in aMCI subjects to confirm that they had an intermediate likelihood of AD neuropathology and in HE, not including those with significant medial temporal lobe atrophy.

All participants gave their written informed consent to participate in the study, approved by the local Ethics Committee of the Santa Lucia Foundation. All procedures performed in this study were in accordance with the 1964 Helsinki declaration and its later amendments or comparable ethical standards.

### 2.2. Study Design

**Figure 2:**
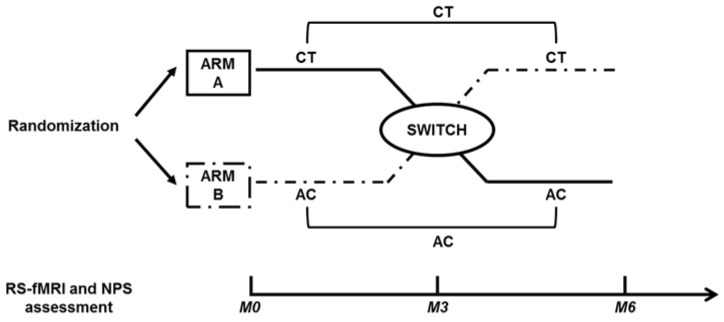
Crossover design. Schematic representation of the study design. All participants received both CT and AC but in a different order: after randomization in the two arms of the study (arm A, arm B). Participants in arm A received first CT and then AC, and in arm B first AC and then CT. Neuroimaging and cognitive outcomes were acquired at baseline (M0), after three months at the switch between treatments (M3), and after six months at the end of the study (M6). Outcomes acquired pre-CT and post-CT and pre-AC and post-AC of both arms were grouped before the statistical analyses.

This was a longitudinal study with a concealed [50] computer-generated simple randomization procedure [51] stratified by sample (mAD, aMCI, HE) that followed a crossover design with two arms: arm A, in which participants first underwent the CT period and then the AC period; arm B, in which participants first underwent the AC period and then the CT period. Both NPS and MRI outcomes were collected at three time-points: baseline (M0), after about three months (i.e., at the switch point between the two periods; M3), and after about six months (i.e., at the end of the study; M6) (Figure 2). The crossover design was adopted in order to allow all participants to receive all treatments and to reduce the effect of confounding variables, having each participant serving as his or her own control. Moreover, in the context of a single center study the crossover design offered a solution to obtain statistical efficiency with a restricted number of participants.

### 2.3. Cognitive Treatment and Active Control

The CT consisted of 24 one-hour sessions twice a week over a period of about three months. CT exercises were in Italian and were administered through a touch-screen computer (see [32] for a full description of the exercises and Appendix A (Table A1) for new exercises added for this study) designed within the SOCIABLE Project [52]. During each session of CT, participants executed for 40% of the total time exercises based on memory (e.g., hiding and finding objects in a room, remembering a menu, learning the association between a face and a name), for 40% of the total time exercises based on executive functions, attention, and reasoning (e.g., abstracting rules, completing logical grids, selecting information among distracters), and for the remaining 20% of the total time exercises based on the remaining cognitive domains (i.e., language, constructional praxis, and orientation). For each exercise, three progressive difficulty levels were provided, adjustable according to the user’s performance, and the participants always started with the easiest level. After two sessions obtaining the maximum score, the difficulty level was increased. This was done in order to achieve the optimal balance between a training-promoting neuroplasticity, i.e., adapted to the user’s performance, and to avoid frustration in the fragile population involved in this study. The requirement of two sessions completed with maximum score was decided based on the results of our previous study [32], which clearly showed that the achievement could be a chance result not reflecting the real ability of the subject. Moreover, increasing the level of difficulty after only one session with the maximum level could generate frustration.

The AC consisted of 24 one-hour sessions held twice a week over a period of about three months. It consisted of entering lists of Italian names and numeric values taken from an Italian lexicon [53] in the same computer used for the CT. This activity was chosen instead of a waiting list or cognitive behavioral therapy [54], in order to control for the time intensity and the setting of the CT [55].

An experienced therapist directed all CT and AC sessions.

### 2.4. Cognitive Outcomes

An experienced neuropsychologist, blind to the randomization of participants, conducted all NPS assessments at M0, M3, and M6. This comprised tests assessing memory, executive functions and attention. Verbal memory was assessed with the RAVLT [34], visual–spatial memory with the ROCF [35], executive functions with the Phonological Word Fluency test [34], the Clock Drawing Test [39], the TMT B-A [36], and attention with the TMT-A [36], the Digit Span Forward [37], and the Attentive Matrices [38].

Raw scores were converted into *z*-scores using means and standard deviations of a healthy elderly sample taken from the Italian standardizations aforementioned, if available; otherwise, we used those derived from our sample of healthy controls. This was done to obtain three average *z*-scores averaging tests assessing the same domain: memory, executive function, and attention. For memory, we averaged the delayed recall *z*-score at RAVLT, the primacy *z*-score at the immediate recall of the same test (obtained taking into account the number of words recalled in the first four positions of the immediate recall test), and the *z*-score at the delayed recall at ROCF. For attention, we averaged the *z*-score at TMT-A, the *z*-score at Digit Span Forward and the *z*-score at Attentive Matrices. Finally, for executive functions we averaged the *z*-score at Phonological Word Fluency test, the *z*-score at Clock Drawing Test and the *z*-score at TMT B-A. For each cognitive domain we computed ΔCT by subtracting the post-CT from the pre-CT and the ΔAC by subtracting the post-AC from the pre-AC.

### 2.5. RS-fMRI Outcomes

#### 2.5.1. RS-fMRI Image Acquisition

MRI scans were acquired on a 3.0 Tesla system (Siemens Magnetom Allegra, Siemens Medical Solutions, Erlangen, Germany). The MRI protocol comprised: (1) 220 volumes of T2* weighted echo-planar imaging (EPI) sensitized to blood-oxygenation-level-dependent (BOLD) (repetition time TR = 2080 ms, echo time TE = 30 ms, 32 axial slices parallel to AC-PC line, matrix = 64 × 64, pixel size = 3 × 3 mm^2^, slice thickness = 2.5 mm, flip angle = 70°) collected at rest for a period of 7 min and 20 s. During this acquisition, participants were asked to keep their eyes closed, not to think of anything in particular, and not to fall asleep; (2) high-resolution 3D modified driven equilibrium Fourier transform (MDEFT) T1-weighted acquisition (TR = 1338 ms, TE = 2.4 ms, Matrix = 256 × 224 × 176, in–plane FOV = 250 × 250 mm^2^, slice thickness = 1 mm); (3) dual-echo turbo spin echo (TSE, TR = 6190 ms, TE = 12/109 ms, same matrix and FOV as the TSE) structural scan; (4) fast fluid attenuated inversion recovery (FLAIR, TR = 8170 ms, TE = 96 ms, inversion time [TI] = 2.100 ms, matrix = 256 × 192; FOV = 230 × 172.5 mm^2^) structural scan. Head movements were reduced during acquisition with cushioning, and headphones were used to reduce scanner noise. Structural acquisitions were reviewed by a neuroradiologist in order to exclude the presence of incidental abnormalities (e.g., clinically significant cerebral vascular lesions).

#### 2.5.2. RS-fMRI Images Pre-Processing

The RS-fMRI pre-processing was done using MATLAB 7.5 R2007B (MathWork, Natick, MA, USA), SPM8 [56], and custom scripts [57]. After discarding the first four volumes of each time series to allow for signal equilibration effects, all images were corrected for acquisition delay between slices, using the second slice as a reference, and for the head motion using a least squares approach and a six-parameter spatial transformation. We estimated the absolute magnitude of the net geometrical displacement vector by combining the three axis components obtained from the preprocessing, and we calculated the displacement between consecutive scans with the absolute magnitude of the first derivative of the displacement vector. The average absolute displacement of all groups was below 0.11 mm and in a 3 × 3 mixed ANOVA (time x group) we found no significant main effects of time or group and no significant interaction. After motion correction, each individual structural image and all functional images were co-registered with the mean EPI image at T0 with the purpose of managing longitudinal data. The transformed structural images were then segmented into gray matter (GM), white matter (WM), and cerebrospinal fluid (CSF) by using a unified segmentation algorithm [58]. Gray matter (GM), white matter (WM), and cerebro-spinal fluid (CSF) probability images were used to compute every subject’s total GM volume (GMV) and intra cranial volume (ICV) and then to compute the GM fraction (GMF), GMF = GMV/ICV. All data were spatial normalized to the Montreal Neurological Institute (MNI) template (resampling voxel size = 2 × 2 × 2 mm^3^) and then spatially smoothed with an 8-mm full-width at half-maximum (FWHM) Gaussian kernel. Data were filtered using a phase-insensitive band-pass filter (pass band 0.01–0.08 Hz) to reduce the effects of low frequency drift and high frequency physiological noise. Finally, for connectomics analyses the resulting signal was fitted with a third-order polynomial fit to remove the contribution of the global temporal drift (detrend), and the realignment parameters and the signal of WM and CSF were averaged over whole brain voxels [59].

### 2.6. DMN Extraction

RS-fMRI images underwent, after pre-processing, spatial independent component analysis (ICA) for DMN extraction. The Group ICA of fMRI Toolbox (GIFT) [60], was used for ICA analysis. ICA maps were estimated using the whole sample of participants (mAD, aMCI and HE) and all three time points (M0, M3, and M6). The GIFT toolbox first concatenated each subject’s data across time and then produced a computation of the specific components and time courses of each subject. The toolbox then performed a three-step analysis as follows: (1) data reduction; (2) application of the FastICA algorithm; and (3) back-reconstruction for each subject [61]. The resulting maps were converted into *z*-scores. Finally, we viewed the 20 resting-state components in order to identify the Default Mode Network (DMN) [21] for each subject and time point. For each subject we computed ΔCT DMN map by calculating the difference between post-/pre-CT DMN maps and ΔAC DMN map by calculating the difference between post-/pre-AC DMN maps.

### 2.7. Construction of Connectivity Matrices

For each participant, we used the information from RS-fMRI to build a matrix representing the whole brain functional network [26]. In each network, the nodes consisted of the 116 brain regions of the AAL atlas [62] co-registered using FSL (FMRIB, Oxford, UK) and for each we calculated the mean BOLD time series across all the voxels within the region. The edges (representing the functional relationships between regions) were the Pearson’s correlation coefficient between these time series of data for each possible pair of regions, and we arranged such correlation values in a 116 × 116 matrix. The choice of correlation as a functional connectivity measure was based on its simplicity and versatility as well as on its wide use in the current literature [63].

We used two approaches to compare the effects of each intervention, CT or AC, on brain networks. The first approach was based on graph theory [27]: we extracted indices describing global and local topological properties of the network and compared them before and after the intervention. The second approach was network-based statistics (NBS) [25] which consists of the comparison between connectivity matrices before and after either CT or AC. The elements of the connectivity matrices quantify the strengths of connectivity between each pair of nodes of the network.

### 2.8. Graph Analyses: Extraction of Local and Global Metrics

From the connectivity matrices of each subject, to compute graph analyses, we built undirected binary matrices representing the absence or presence of functional coupling [64]. Since the choice of a single threshold could lead to unreliable results [63], we used a multiple threshold approach implemented in MATLAB with custom scripts: for each subject, we selected several thresholds in order to obtain fixed density values, where the density is the ratio between the number of existing connection and the number of all the possible connections. This approach could prevent spurious results that could be only attributed to differences in density [64]. We chose the density range starting and ending points in order to avoid isolated nodes (i.e., disconnected regions) but still keep a small-world architecture (i.e., avoiding random topology).

We computed graph measures using Brain Connectivity Toolbox (BCT) [65] and in-house Matlab scripts in order to explore both the global and local topological properties of the brain network of each participant [27]. Several indices can be derived from graph theory [64], but for the purpose of the present study we focused on indices with a more clinical interpretation and most commonly investigated in previous studies [66,67]. We calculated for each subject the mean clustering coefficient, the characteristic path length and the small-worldness describing global properties and degree, betweenness centrality and local efficiency describing local properties. The mean clustering coefficient is a measure of functional segregation of the network and it reflects the clustered connectivity around nodes. The characteristic path length measures the functional integration of the network and it is the average of the shortest path lengths between all pairs of nodes. In order to avoid spurious results, we normalized global metrics evaluating the ratio between the original metrics and the ones retrieved from random networks (100 random networks obtained rewiring the original ones keeping the original degree distribution). In order to characterize the network architecture, we also calculated the small-worldness, which is the ratio between the normalized mean clustering coefficient and the normalized characteristic path length. The degree, betweenness centrality and local efficiency of a node reflect its centrality, i.e., its importance in serving as a hub. The first represents the number of the nodes connected to a given node, while the second is the fraction of all shortest paths in the network passing through a given node. Finally, the last is the ratio between the number of connections of a node with its neighbors and the total possible connections.

### 2.9. Statistical Analyses

For the analyses of NPS and RS-fMRI outcomes we considered as CT period (pre- and post-CT) both the one of arm A and B and the same for the AC period (pre- and post-AC) both the one of arm A and B (Figure 2).

We performed the following statistical analyses for the different outcomes:

(1) Cognitive: for each cognitive ability (memory, executive functions, attention) we compared the ΔCT with ΔAC for all subjects together and then separately for each sample (mAD, aMCI, and HE). Due to the limited sample sizes and the skewed distribution of some of the variables, we used non-parametric test of Wilcoxon. For baseline comparisons (Table 1), we used the Mann–Whitney U test to compare the two arms of the study (A vs. B) and test statistic of Kruskal–Wallis to compare the three groups (mAD, aMCI, and HE). We accepted significance levels of *p* < 0.05.

(2) DMN: according to previous evidence on the impact of cognitive reserve in the DMN of AD patients [57], we tested for differences in the spatial extend of the DMN rather than its temporal integrity. We compared the ΔCT DMN maps with ΔAC DMN maps in a voxel-wise whole-brain second level flexible factorial design with a 2 × 3 ANOVA with treatment (i.e., ΔCT or ΔAC) and group (i.e., mAD, aMCI, HE) as factors. We tested the main effects and the interactions. Age, education and the gray matter fraction (gray matter to the total intracranial volume ratio, a measure of atrophy) were entered as covariates of no interest to adjust the analysis for potential confounds. We also compared the three groups for differences at baseline with a voxel-wise whole-brain second level full factorial design. Correction for multiple testing was accounted for by accepting the results of the main effects and their interactions as significant at *p*-values < 0.05 FWE corrected at cluster-level, with an uncorrected threshold of *p* < 0.005 at the voxel level. Finally, we extracted the neuroimaging data for CT (ΔCT beta values of the main peaks) for significant results. Then, we calculated, separately for each group, the correlation between the ΔCT of functional neuroimaging data for significant results for that group with ΔCT of behavioral data for memory, executive functions and attention with a Spearman’s rank correlation.

(3) Connectomics analyses: for each of the graph theory metrics (i.e., mean clustering coefficient, characteristic path length, small-worldness, degree, betweenness centrality, local efficiency) we tested the differences between post-CT vs. pre-CT and separately the differences between post- vs. pre-AC separately for each sample (mAD, aMCI and HE). We assessed significant differences using the non-parametric permutation test (1000 permutation) corrected by false discovery rate (FDR, *p* < 0.05) in order to take into account multiple comparisons [68]. We used the area under the curve (AUC) of each metric in order to sum up the results over the density range [63].

Network-Based analyses. We investigated the differences between all pairs of regions between post- vs. pre-CT and separately post- vs. pre-AC separately for each sample (mAD, aMCI and HE) in terms of functional subnetworks using NBS Connectome software package [25]. This approach involves three steps. The first consists in a mass univariate testing consisting in the independent test of each connection with a test statistic (in this study a *t*-test) between post- and pre-treatment. The second step consists in calculating the size of the largest component (i.e., a subsample of connections) exceeding a primary threshold (in this study *t* = 3.1). Finally, as the third and main step, we computed the *p*-value for each connection using a non-parametric permutation-testing procedure (here we used 10,000 permutations). In this procedure, the size of the largest components is re-computed for each of the 10,000 random permutations of group assignments (pre- vs. post-treatment). The result was controlled for multiple comparison with false discovery rate procedure (FDR) with *p* < 0.0.05.

For nodes/edges that showed significant changes after CT, we extracted the parameters for both pre-CT/post-CT and pre-AC/post-AC. We calculated the differences between post-/pre-CT (ΔCT) and between post-/pre-AC (ΔAC). We tested the difference between ΔCT and ΔAC with a non-parametric test of Wilcoxon for each significant node or edge. We also correlated ΔCT of connectomics parameters of significant nodes/edges for each group of participants with their ΔCT of cognitive data for memory, executive functions, and attention with a Spearman’s rank correlation.

## 3. Results

### 3.1. Cognitive Outcomes

Table 1 shows groups’ characteristics at baseline. The two arms of the study (A vs. B) showed no differences in demographic characteristics and cognitive scores. The three samples performed differently at tests for memory, attentional, and executive functions.

Table 2 shows the behavioral results comparing ΔCT vs. ΔAC. When analyzing all subjects together, we found a significant difference in memory Δ*z*-scores, showing a larger positive effect after CT vs. after AC (*p* < 0.05) that was mainly driven by aMCI (*p* < 0.05). We also found in all subjects a significant difference in attention Δ*z*-scores, showing a larger positive effect after CT vs. AC (*p* = 0.01) that was mainly driven by mAD patients showing an almost significant effect (*p* = 0.07).

### 3.2. Neuroimaging Outcomes

#### 3.2.1. DMN Analyses

**Figure A1:**
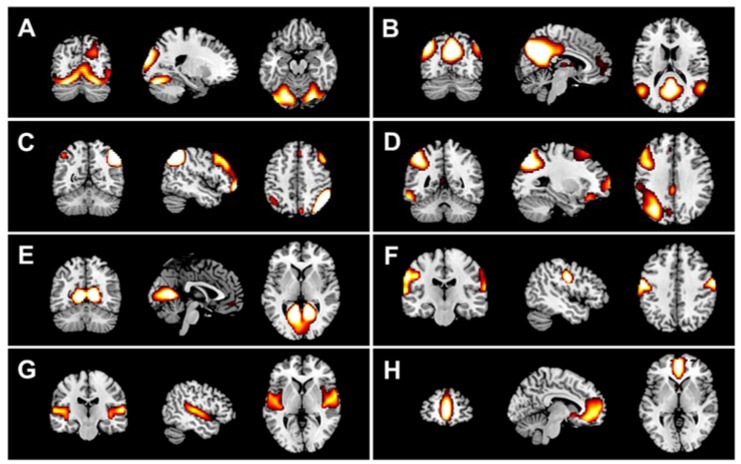
Resting-state networks. The figure shows the resting-state networks extracted with the ICA analysis consistently to previous studies [21]. They include the extra-striate visual network (**A**); the default mode network (**B**); the right and left lateralized networks, consisting of superior parietal and superior frontal regions (**C**,**D**); the primary visual network (**E**); the primary sensorimotor network (**F**); the network consisting of bilateral temporal/insular regions (**G**) and the network consisting of the anterior cingulate cortex regions (**H**).

ICA analysis isolated the DMN including the bilateral posterior medial, lateral parietal cortices and, to a lesser extent, the medial prefrontal cortex bilaterally, as shown in Appendix A (Figure A1).

At baseline (Table 3), mAD showed decreased functional connectivity in the posterior node of the DMN (the bilateral precuneus) compared to aMCI. The latter showed greater functional connectivity compared to HE in the precuneus and the left postcentral gyrus. MTA was on average 2.05 (SD 0.65) for mAD, 1.5 (SD 0.4) for aMCI and 0.43 (SD 0.47) for HE.

**Figure 3:**
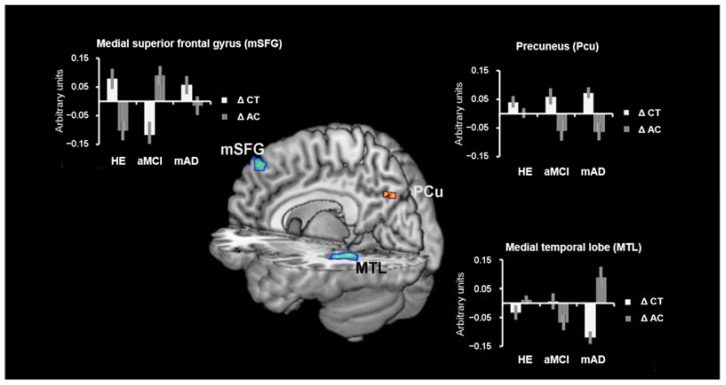
Changes in DMN. Left panel: Effect size of mSFG (region in blue in the left central rendering) functional connectivity changes of the significant interaction between aMCI and HE and treatment, showing a greater functional connectivity decrease in aMCI vs. HE after the ΔCT (white columns) vs. ΔAC (lined columns). Right top panel: Effect size of PCu (region in hot color in the central render) functional connectivity changes, showing the significant main effect of the whole sample of participants between ΔCT (white columns) > ΔAC (lined columns). Right bottom panel: Effect size of the MTL (region in blue in the right central rendering) functional connectivity changes, showing the significant main effect in mAD sample between ΔCT (white columns) < AC (lined columns). Abbreviations: ΔCT = cognitive training; ΔAC = active control condition; HE = healthy elderly; aMCI = amnestic Mild Cognitive Impairment; mAD = mild Alzheimer’s Disease; mSFG = medial superior frontal gyrus; PCu = precuneus; MTL = medial temporal lobe.

The two-way ANOVA (treatment × group interaction; Table 3 and Figure 3) revealed a significant positive main effect of CT treatment (ΔCT > ΔAC) in the whole sample of participants in the posterior node of the DMN, the right precuneus. We also observed a significant interaction between aMCI and HE ((aMCI ΔCT < aMCI ΔAC) > (HE ΔCT < HE ΔAC)) in the bilateral medial superior frontal gyrus (mSFG), showing, after CT, a decreased functional connectivity for aMCI vs. an increased functional connectivity for HE.

Stratifying these contrasts by group, we observed that the main effect (ΔCT > ΔAC) was significant only for mAD in the same region (*x* = 10, *y* = −70, *z* = 38 (*t* = 3.96, *p*-FWE < 0.01)). Moreover, mAD showed a significant decrease of functional connectivity after CT (ΔCT > ΔAC) of the left posterior medial temporal lobe (MTL). No significant correlations emerged between functional connectivity and cognitive changes.

#### 3.2.2. Connectomics Analyses

At baseline, on global measures (i.e., clustering coefficient, characteristic path length, small-worldness) mAD showed decreased small-worldness compared to aMCI (*p* < 0.05) and a decreased nodal degree of the left anterior cingulum compared to HE (*p*-unc. = 0.001).

**Figure 4:**
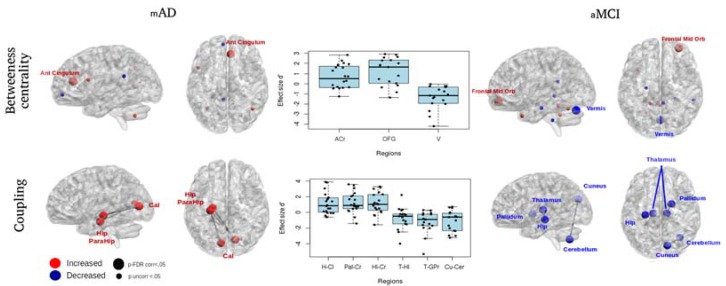
Changes in connectomics. Lateral panels. Significant changes after CT in betweenness centrality (top row) and in coupling strength between couples of nodes (bottom row) in mAD patients (left panel) and subjects with aMCI (right panel). In red are the increases and in blue are the decreases of the metrics. Central panel. Scatter plots with effect size of individual subjects superimposed calculated effect by dividing the difference between ΔCT and ΔAC by their pooled standard deviation [69], for significant regions and edges. Abbreviations: mAD = mild Alzheimer’s disease; aMCI = Mild Cognitive Impairment; CT = cognitive training; l = left hemisphere; r = right hemisphere; AC = anterior cingulum; OFG = Orbito frontal gyrus; V = vermis; H = hippocampus; Pa = parahippocampal gyrus; C = calcarine cortex; T = thalamus; GP = globus pallidus; Cu = cuneus; Ce = cerebellum.

Graph analyses (see Table 4; Figure 4) showed only after CT: in aMCI there was a significant increase of the betweenness centrality of the right orbito-frontal region and a decrease in the vermis of the cerebellum; in mAD there was a significant increase in the betweenness centrality of the right anterior cingulum. No significant results emerged after AC and for HE.

NBS analyses showed only after CT in aMCI there was a decreased functional connectivity between the thalamus and the hippocampus in the left hemisphere and in the right hemisphere between the thalamus and the globus pallidus and between the cerebellum and the cuneus; in mAD there was an increased connectivity between both calcarine cortices and the left medial temporal lobe. No significant results emerged after AC and for HE.

Significant differences between ΔCT and ΔAC emerged for all the nodes/edges showing changes after CT (Table 4). No significant correlations emerged between the cognitive data and the connectomics data.

## 4. Discussion

The present study showed that a period of a computerized CT mainly focused on memory and executive functions promoted improvements in memory and attention. These came along with functional connectivity changes in the brain networks of patients with mAD and in subjects during its preclinical phase of aMCI.

Our cognitive results replicated our previous study [32] showing memory improvement after CT, particularly in the aMCI sample, also when these were compared to an active control condition. Conversely, mAD showed mainly attentional improvements after CT. These findings agree with a recent meta-analysis [16] showing that computerized CT is efficacious on select cognitive domains in people with MCI rather than AD patients, where efficacy is less consistent.

Our neuroimaging results showed two different patterns of results for mAD and aMCI. CT modulated mainly regions within the DMN in mAD, whereas aMCI showed widespread topological changes involving cortical and subcortical brain structures.

Our mAD sample showed functional connectivity changes between key regions for memory processes that are affected by AD pathology [70] such as the precuneus, the anterior cingulum, and the medial temporal lobe. All these regions are part of the DMN, which is critical for both AD progression [3,4,5] and brain resilience to disease [57]. However, AD involves the alteration of distributed networks of brain regions, renewing research interest in considering AD in part as a disconnection syndrome [71]. Although the current evidence on AD pathology is far from conclusive [72], this disease may begin within key regions central for the network called ‘hubs’, which are preferentially vulnerable to neuropathology [73]; their alteration determines consequences within the network architecture [74]. In fact, consistent with previous evidence in people at risk of developing AD [5], we showed a CT-related increase of functional connectivity within the DMN in the precuneus, but we also showed the increase of the anterior cingulum centrality when we extended our analysis to the topological organization of the entire brain network. Indeed, the same region showed at baseline reduced functional connectivity in mAD and, consistent with previous evidence [29], the same group showed reduced small-worldness. Two previous pharmacological trials in AD [75,76], using functional connectivity at rest as outcome measure, showed increased functional connectivity in the medial prefrontal regions after a period of treatment with dopenezil. Moreover, a pioneering study using graph theory in MCI [31] showed that an intervention of combined physical and CT induced functional connectivity changes involving nodes connected to the DMN. Consistently, we found some effects in local topological properties (i.e., betweenness centrality) that quantify the centrality of a region. Nodes with high centrality, or hubs, highly interconnected to the rest of the network, play a critical role in integrative and compensatory processes. Their damage has a high impact on the entire network [73] and could be related to the decrease of brain metabolism consequent to the disease [77].

Our mAD sample also showed CT-related functional connectivity changes of the medial temporal lobe. In particular, we found decreased functional connectivity in this region when the analysis was restricted to the DMN, and increased functional connectivity between the medial temporal lobe and occipital regions when the analysis was extended to the entire brain in our NBS analyses. Increased temporo-occipital functional connectivity was also shown in a previous study with MCI [20] after a period of CT based on face–name associations. The current interpretation of these effects might be only speculative considering the lack of significant correlations between our neuroimaging and cognitive data. The increased temporo-occipital functional connectivity showed in mAD patients might constitute an attempt to recover their decreased functional connectivity between the temporal lobe and the parietal and occipital cortices [78] that might explain the visual attentional and encoding deficits of AD patients [79]. Conversely, the decrease in the medial temporal lobe functional connectivity within the DMN might be an attempt to restore brain organization by reducing inefficient connections [80]. However, further investigations are needed to be conclusive.

Our aMCI sample showed complex CT-related effects involving several brain regions, as reported in previous task-based fMRI studies in MCI [19]. Consistent with previous evidence [22], our aMCI sample showed CT-related changes of the functional connectivity within the DMN. Extending our analysis to the whole brain, we found decreased thalamic connectivity with the medial temporal lobe and the basal ganglia. We also observed CT-related changes in the centrality of the vermis and the orbito-frontal region. Previous studies suggested increased functional connectivity in MCI within the DMN [81], the thalamus [82], and the cerebellum [9]. Consistently, at baseline our aMCI patients also showed increased functional connectivity within the DMN. All these regions are involved in memory processes [83,84] and it has been suggested that increasing functional connectivity may represent a maladaptive reorganization process contributing to the memory deficit in MCI [81,85]. Although the mechanisms underlying the CT modulation of connectivity are not yet fully understood, we speculate that CT might counterbalance this initial compensation mechanism of increased connectivity, as has been postulated for the healthy elderly [86]. However, considering the lack of correlation with cognitive results, this might be too simplistic a speculation that needs further studies [9].

Although several studies reported CT-related effects in the healthy elderly [17], our HE group showed no functional connectivity effects. Our aim was to test the effects of a CT focused on memory exercises in patients with memory disorders. Indeed, we enrolled the HE as a control group and the lack of CT effects in HE was expected since they were carefully selected to perform in the normal range in all cognitive tests. Thus, the effects of CT might be minimal.

The weaknesses of the present pilot study include the limited size of the samples. This might explain the lack of significant correlation between cognitive and neuroimaging measures or even the lack of a significant effect in HE. It should be noted that we correlated changes in complex brain networks and changes in complex cognitive functions and the null effect in our brain–behavior analyses might be due to the complexity of the measurement that we considered. Moreover, although the crossover design requires fewer subjects to obtain statistical efficiency, a parallel study is advocated to control for confounding effects. For this purpose, a large, multicenter, randomized controlled trial is warranted to corroborate the present results and evaluate the clinical relevance of CT. Moreover, other potential confounding effects in fMRI studies of neurodegenerative diseases are related to differences in vascular signals [87]. The use of generative models might allow us to distinguish age and disease effects on the true neural connectivity from changes in the neurovascular coupling [88].

## 5. Conclusions

In conclusion, our study supports the selective effect of CT to modulate whole-brain functional connectivity in subjects at risk of developing AD and in patients during the early stages of this disease. Moreover, it also highlights the different effects of CT between preclinical and clinical stages of AD. However, due to the pilot nature of the present study and the lack of correlation with cognitive data, further studies are needed to better understand the brain plasticity effects of CT and to develop effective forms of intervention specific to each level of severity of AD.

## Figures and Tables

**Table 1:** Average values (SD) for demographic variables and cognitive performance at baseline.

	mAD (*n* = 22)				aMCI (*n* = 23)				HE (*n* = 25)				All	
	Arm A (*n* = 14)	Arm B (*n* = 8)	*Χ*^2^/U	*p*	Arm A (*n* = 10)	Arm B (*n* = 13)	*Χ*^2^/U	*p*	Arm A (*n* = 12)	Arm B (*n* = 13)	*Χ*^2^/U	*p*	*Χ*^2^/H	*p*
Sex (male/female)	5/9	3/5	0.007	0.993	7/3	7/6	0.619	0.431	4/8	3/10	0.326	0.568	5.673	0.059
Education (year)	9.6 (3.7)	10.1 (3.2)	50	0.714	10.7 (3.1)	12.2 (4)	50	0.376	11.2 (4.2)	11 (4.2)	79	1	2.63	0.268
Age (year)	76.4 (6)	73.9 (4.7)	64	0.616	71.4 (6.6)	72.8 (5.7)	53	0.446	69.9 (5.6)	71 (6.8)	70	0.650	7.15	0.028
Memory (*z*)	−2 (0.5)	−1.6 (0.63)	30	0.082	−0.67 (0.65)	−0.87 (0.67)	73	0.648	0.44 (0.93)	0.18 (0.96)	89	0.547	42.98	<0.001
Attention (*z*)	−0.87 (1.25)	−0.10 (1.05)	38	0.238	0.76 (0.78)	0.71 (0.73)	72	0.693	1.05 (0.43)	1.36 (0.36)	47	0.098	28.02	<0.001
EF (*z*)	−0.27 (0.89)	−0.79 (1.03)	70	0.365	0.15 (0.75)	−0.03 (0.86)	73	0.648	0.57 (0.61)	0.61 (0.52)	84	0.769	15.95	<0.001

Abbreviations: SD = standard deviation; HE = healthy elderly; aMCI = amnestic mild cognitive impairment subjects; mAD = mild Alzheimer’s Disease patients; EF = executive functions; *p* = 2-sided probability value; U = test statistic of Mann–Whitney U test comparing the two arms A vs. B; H = test statistic of Kruskal–Wallis test comparing the three groups; *z* = *z*-scores calculated on published normative data of the same age and education sample.

**Table 2:** Behavioral results. Mean *z*-score (SD).

	ALL						mAD						aMCI						HE					
	ΔCT	ΔAC	T	*p*	*N*	*r*	ΔCT	ΔAC	T	*p*	*N*	*r*	ΔCT	ΔAC	T	*p*	*N*	*r*	ΔCT	ΔAC	T	*p*	*N*	*r*
**Memory (*z*)**	0.30 (0.54)	0.10 (0.55)	903	**0.05**	70	0.17	0.14 (0.36)	−0.14 (0.51)	81	0.14	22	0.22	0.32 (0.47)	0.03 (0.51)	73	**0.05**	23	0.29	0.42 (0.7)	0.38 (0.5)	158	0.9	25	0.02
**Attention (*z*)**	0.20 (0.65)	−0.20 (0.74)	778	**0.01**	70	0.23	0.28 (0.94)	−0.47 (1.14)	71	0.07	22	0.27	0.21 (0.42)	−0.01 (0.46)	92	0.16	23	0.21	0.12 (0.5)	−0.14 (0.38)	103	0.11	25	0.23
**EF (*z*)**	0.08 (0.66)	0.05 (0.6)	1093	0.38	70	0.07	−0.06 (0.82)	0.12 (0.79)	129	0.94	22	−0.01	0.04 (0.66)	0.01 (0.6)	129	0.78	23	0.04	0.24 (0.45)	0.03 (0.39)	112	0.17	25	0.19

Abbreviations: SD = standard deviation; ALL = all participants; HE = healthy elderly; aMCI = amnestic mild cognitive impairment subjects; mAD = mild Alzheimer’s Disease patients; EF = executive functions; ΔCT = difference between post-CT and pre-CT; ΔAC = difference between post-AC and pre-AC; *p* = probability value 2-sided; T = test statistic of Wilcoxon signed-rank comparing the ΔCT and ΔAC (significant *p*-values are in bold); *z* = *z*-scores calculated on published normative data of the same age and education sample; *N* = number of subjects in the Wilcoxon signed-rank test analysis; *r* = effect size derived from T statistic converted in *z*-scores/root square of the number of total observations.

**Table 3:** Analysis of DMN functional connectivity at baseline and comparing ΔCT vs. ΔAC.

Contrast	Sample	H	Region	Cluster (voxel)	*t*	FWE_corr *p* Cluster	MNI Coordinates		
							*x*	*y*	*z*
**Baseline**	**aMCI > HE**	L	Postcentral gyrus	360	4.98	0.014	−34	−20	40
		L	PCu	1253	4.63	<0.001	−2	−46	50
		R	PCu		4.62		10	−60	34
	**mAD < aMCI**	L	PCu	610	4.79	<0.001	−4	−70	28
		R	PCu		3.97		10	−62	28
**ΔCT > ΔAC**	**ALL groups**	R	PCu	288	4.22	0.033	14	−64	48
**ΔCT < ΔAC**	**mAD**	L	MTL	302	5.21	0.026	−28	−42	−4
**INT**	**aMCI (ΔCT < ΔAC) > HE (ΔCT < ΔAC)**	R	mSFG	288	4.56	0.033	2	46	44
		L	mSFG		3.93		−8	50	30

Abbreviations: H = hemisphere; L = left; R = right; CT = cognitive training; AC = active control condition; HE = healthy elderly; aMCI = Mild Cognitive Impairment; mAD = mild Alzheimer’s Disease; mSFG = medial superior frontal gyrus; PCu = precuneus; MTL = medial temporal lobe.

**Table 4:** Connectomics results. Mean metrics/edges values (SD).

**Method**	**Group**	**CT Effect**	**Measure**		**Brain Area**	**Side**	**ΔCT (Post-Pre)**	**ΔAC (Post-Pre)**	**T**	***p* (2-Tailed)**	***N***	***r***
BCT	mAD	(post CT > pre CT)	Betweenness centrality		Anterior cingulum	R	8.26 (10.66)	−0.91 (14.46)	51	0.044	20	0.32
	aMCI	(post CT > pre CT)	Betweenness centrality		Orbito-frontal region	R	7.77 (5.25)	−2.39 (8.52)	21	0.009	17	0.45
	aMCI	(pre CT > post CT)	Betweenness centrality		Cerebellum-Vermis		−10.7 (10.28)	6.61 (8.53)	155	<0.001	17	−0.62
**Method**	**Group**	**CT Effect**	**Edge**				**ΔCT (Post-Pre)**	**ΔAC (Post-Pre)**	**T**	***p* (2-Tailed)**	***N***	***r***
			**Brain Area**	**Side**	**Brain Area**	**Side**						
NBS	mAD	(post CT > pre CT)	Calcarine cortex	L	Hippocampus	L	0.19 (0.23)	−0.16 (0.27)	21	0.002	20	0.5
			Calcarine cortex	R	Parahippocampal gyrus	L	0.22 (0.23)	−0.16 (0.27)	17	0.001	20	0.52
			Calcarine cortex	R	Hippocampus	L	0.19 (0.18)	−0.11 (0.26)	22	0.002	20	0.49
	aMCI	(pre CT > post CT)	Thalamus	L	Hippocampus	L	−0.17 (0.17)	0.05 (0.34)	122	0.031	17	−0.37
			Thalamus	R	Globus pallidus	R	−0.17 (0.18)	0.13 (0.18)	144	0.001	17	−0.55
			Cerebellum	R	Cuneus	R	−0.2 (0.2)	0.13 (0.29)	130	0.011	17	−0.43

Abbreviations: aMCI = amnestic mild cognitive impairment subjects; mAD = mild Alzheimer’s Disease patients; CT = cognitive training; AC = active control condition; L = left; R = right; T = T statistic for the Wilcoxon test comparing ΔCT and ΔAC of the same subjects; *p* (2-tailed) = *p*-value 2-tailed for the Wilcoxon test; *N* = number of subjects in the Wilcoxon test analysis; *r* = effect size derived from T statistic converted in *z*-scores/root square of the number of total observations.

## Appendix A

**Table A1:** New exercises added to the SOCIABLE CT training [32].

Exercise	Contents	Objective of Training
	Memory	
Person–name learning	The user is presented with pictures of people associated with a name and is asked to memorize their names. After a distracting visual–spatial attentional task, the same pictures are represented but their names might be correct or switched with one of the others. The user has to indicate whether the name associated with each face is correct or not.	Declarative episodic long-term visual–spatial memory
	Executive functions	
Remember the sequence.	The user is asked to remember an increasing sequence of images placed on an *n* × *n* grid forming a path. At the same time, he must press a button each time an item from a target category is presented (e.g., animals).	Working memory
